# Seventh Annual Session of the Maryland State Dental Association

**Published:** 1890-07

**Authors:** 


### Reports of Society Meetings.

### SEVENTH ANNUAL SESSION OF THE MARYLAND STATE DENTAL ASSOCIATION.

(Continued from page 296.)

### DISCUSSION OF DR. GIBSON'S PAPER ON "OBTURATORS FOR ACQUIRED AND CONGENITAL LESIONS OF THE HARD AND SOFT PALATE."

*John N. Mackenzie, M.D.*, of Baltimore.—Mr. President, I came here to listen and to learn, not to speak,—a fact which will explain the reluctance with which I rise to respond to the kind invitation of the chair. I have been much interested in the able paper of Dr. Gibson. Permit me here, in advance, to say that my friend, Dr. Keech, is apparently laboring under a misapprehension in regard to the purport of the paper read by me, the other evening, at the meeting of the Hopkins Hospital Society. The subject of that paper was double uvula as contradistinguished from cleft of the soft palate or cleft of the hard and soft palate. The paper purported or was intended to treat of certain forms of this deformity which I had met with in my practice, and which are not noticed in the books. I attempted to refer the anomaly to the absence of the uvula in the lower types of creation, and in that way to connect it with a sort of reversion to the structure of otarian forms. In other words, the subject-matter of my paper was not the surgical treatment of cleft palate.

I was sensibly impressed, this evening, by the remark made by Dr. Gibson, in the initiatory portion of his essay,—viz., that if the appliances to which he referred did nothing else they would create a normal respiration. We all appreciate the value of healthy nasal breathing; and when nasal breathing becomes transformed into mouth breathing, it is a menace to perfect physiological life. The integrity of many structures is thus impaired,—not only the lower respiratory tract, but the middle ear and nasal pharynx, which may be regarded as accessory chambers to the nose. The middle ear may be regarded in that light, because, in many cases, you will find the middle ear diseased to be symptomatic of the nasal trouble, and that it will disappear upon the dissipation of its primary cause. It seems to me that these appliances will be held in those difficult cases in which surgical interference is not justifiable.

I have been much gratified in listening to Dr. Gibson, to learn of the advances that have been made by him in that direction. He may be said to have only furnished other evidence in corroboration of the claim that is made in behalf of American dentistry,—one that is well known to the medical as well as to the dental profession,—that it is in the forefront of progress, and that its fame has reached to the uttermost limits of civilization. That fact is one that is universally recognized, and it is one of which Americans, when abroad, are justly proud.

Returning to the subject of the essay: One inquiry that I would like to propound to Dr. Gibson is this, Why should the obturator be made so large and ponderous? The name would seem to indicate that the contrary is true; but we know it to be a fact that the uvula and the soft palate are lifted up mainly through the primal agency of the tensor palati muscle. It was proven, long ago, by Czermak that, in the production of all oral sounds, whether vowels or consonants, the soft palate is brought up against the posterior wall,—not as a hinge, as in the case related by Dr. Gibson (and therefore failing in the accomplishment of the object), but as a curve. For example, there are certain valves in the heart and in the veins, and the soft palate forms a valve similar to one of those. Moreover, the isolation of the nasal cavities from the mouth in speech is not accomplished by the soft palate alone. It is generally supposed by medical men that the constrictor muscles of the pharynx are all concerned in deglutition. This is a great mistake. The inferior middle and the middle constrictor muscles are the ones concerned in swallowing, while the superior constrictor muscle is one that is conspicuously concerned in the production of speech. During the production of all oral sounds a slight bulging of certain fibres of the superior constrictor takes place; and upon that the soft palate rests. That was demonstrated by Passavant, in a case in which the palate was so high that the fact was capable of ocular demonstration.

Czermak proved the fact of the elevation of the soft palate by introducing wires of various kinds through the nose and having the patient articulate various sounds. It was found that during the articulation, especially of the vowel "I" (the German "T"), the soft palate reached its maximum elevation. That was tested by men like Brücke, Shuh, and a number of others. Shuh had a case in which the whole front—the entire aspect—of the face was removed and the floor of the nostrils laid bare, so that he could watch the elevation of the palate during the articulation of the vowels. Czermak, Brücke, and other distinguished physiologists, corroborated these experiments, and the fact established by them is undoubted.

I would like to know whether observations of that kind upon these artificial palates have been made, and whether, in the production of all purely oral sounds, the cavity of the mouth is isolated from that of the nose. It may happen in this way that, if such isolation is not complete, the dorsum of the tongue may act as a sort of vicarious representative of this muscular action and be shoved farther up. In the production of the nasal vowels you are conscious of two motions,—an upward and backward motion of the tongue and a downward and forward motion of the soft palate. This, of course, cannot be demonstrated by looking in the mouth, but a consciousness of the fact exists. It may be that, in these artificial palates, the dorsum of the tongue may act vicariously in the place of the soft palate. It is also possible that more fibres of the constrictor may be concerned, and that nature may so adjust the superior constrictor that it may also vicariously act in the place of the soft palate.

I regard the question as an exceedingly interesting one, and I am apprehensive that the medical profession, as a rule, fails to recognize the importance of these cases, and is rather disposed to allow the patients to go home as incurable, and to assume that nothing can be done for them without an operation is performed. I think that Dr. Gibson has not only done this society great good, but has rendered the whole medical profession a valuable service by bringing before them the triumphant results of his marvellous ingenuity.

*Dr. Gibson.*—In response to the question, I beg to say that I have failed to find in anything written on this subject any great importance attached as to what the tongue can be made to do in these cases, and, in my opinion, it does play *a very important part.* There is in these cases a separation of the uvula, and as a rule each part is unusually large. The appliance is so adapted as not to interfere with the operations of the muscles of the palate. The two portions of the uvula back up (if I may so express it) against the pharynx, thus closing up all space left by the obturator and constrictor muscles. The velum and uvula also move anteriorly to the obturator.

If the soft-rubber velum were permanent and not so intricate and expensive in its construction, it would without doubt be the best appliance that could be used, as all the muscles of the soft palate are brought into use and the velum is elevated with them. These objections, however, preclude its general use, as the majority who need the appliance are not people of wealth.

These considerations impelled me to turn my attention to hard rubber; and I can say, from what I know in regard to it, that if the length and the proper curve are obtained, with the aid of the tongue and the constrictor muscles and with the moving back and forth of the soft palate, we can accomplish all that it is in our power to do at the present time. I indulge the hope that, in the near future, some one may succeed in finding a substance which will be sufficiently elastic and permanent to admit of it being utilized for the purpose required. I trust that my anticipation in this respect may be realized.

*Dr. D. Genese.*—The present is the third occasion in the United States, during a period of twelve years, upon which I have had the pleasure of seeing so many beautiful pieces of workmanship as are here shown; and I have rarely heard so complete a report in regard to them and such full explanations of practical demonstrations as we have received to-night from Dr. Gibson. Undoubtedly that which is here treated of is one of the chief branches of dental science, and one which is destined to raise the standard of our profession above the level of the mere tradesman. The models exhibited here are for the most part similar in character. The apparatus shows the fissures in the soft palate partly and in the hard palate partly but shows them all posteriorly.

In my own practice I have had a case in which the fissure was in the anterior portion of the mouth. The history of the case is as follows: It originated from a gun-shot wound in the late war. The wound healed and left a small brown spot, which irritated the patient. This the patient had the misfortune to open with a watch-key, and, in so doing, poisoned the wound. Lupus set in, and the result, after a period of thirteen years, was the loss of the entire anterior portion of the face. The case was seen by a number of practitioners in this city, but they objected to handling it because of the difficulty, or what seemed to be the impossibility, in their opinion, of securing a model of the parts because of the large fissure.

Let me here say that on this point I beg to differ from our *confrère*, Dr. Gibson, in the position he has taken, as I contend that a model can be taken of any soft tissue of the human frame, without displacing it and without subjecting the muscles to any tension. The model, however, should be taken in sections,—one, two, or three, or as many as are required,—ample time being allowed, so that the work may remain in the mouth in a plastic state until the muscles have recovered their normal position. You all know that the reflex action of the nerve-centre will cause the muscles to contract; and if you insert a foreign body against the soft tissue, particularly if it is a metal substance, you are going to have contraction there, which, if continued in a permanent piece of work, will end in absorption.

A model of any piece of tissue can be taken with the material used for renovating old composition. This material is very nearly as soft as beeswax, and will become very plastic or almost as plastic as new putty when heated to 180° of temperature.

Should your palate present any undercuts, provide yourself with little pieces of card-board, cut in any form that may be desired. Procure the modelling composition, or, if you prefer it, use plain beeswax. Insert it in one side of the fissure and hold it there for a few minutes with the finger or the instrument. Place a piece of card-board upon the side of it and press it in place. That will remain in position for a while. Then get your patient to close his or her mouth and so keep it in place with the tongue. In the mean time you are preparing another piece with another section of the card-board. The outer surface of the card-board that you have already caused to adhere to the composition you will then oil or soapstone, whichever you prefer. You will then prepare the third section and fill in. But you should take care that you can draw the card-board so as to leave a division by which you can move your last section and draw that out separately.

When you have thus placed your soft palate in, you will let the patient take some hot water in his or her mouth and keep moving it around in the mouth, thus bringing the muscles into play and forcing the substance of this material into something like the position of the muscles when in repose. The lower surface can be so made that the plaster impression that you take will readily be preserved and withdrawn. When the impression is removed and these sections are taken away, any pieces can be replaced and put into this hollow of the plaster impression. In that way you can get a model of almost any case that comes under your notice.

The case to which I was referring was one that ended in lupus, as I have said. I made a model in that case in the manner I have described. I wish to say here that no one who has not experienced it can fully appreciate the sense of comfort which a patient derives when work such as that which Dr. Gibson has shown us this evening is placed in the mouth. I suppose we all have some endearing expression which is peculiar to us, and that is generally the kind of an expression which finds utterance by a patient when he realizes the improvement which is made by this means. In the case of the patient of whom I have spoken, who was a poor man, his first exclamation as he looked up, after the piece was placed in his mouth, was, "Doctor, isn't this hunkidory?" That was the first intelligent expression that he had been heard to utter for a long time. Without the apparatus, he could not articulate at all. I mention this as substantiating what Dr. Gibson has said in regard to having the tongue educated to assist in the work. This patient, if any one came upon him suddenly, would try to hide his deformity by taking a silk handkerchief, throwing it up, catching it with his tongue, and holding it there as though his tongue were a peg. Such is the power that may be acquired over the movements of the tongue by practice. In that case, it was fortunate for me that the wisdom teeth remained and that I was able to attach the plate to them. The front part of the face, the nose, and the lip were all gone. I replaced these parts with white rubber and put on a piece of rabbit-skin to do service in the place of a moustache. The poor fellow then became able to go out in the street, though he had not been out for some years. He resided in this city. Before that apparatus was placed in his mouth he had not been out of his house for five years. The insertion of it enabled him to appear on the public street without being made an object of ridicule. His death, which occurred last year, was not due to any fault or defect of the apparatus, but was simply a natural consequence of the terrible disease of lupus, which had encroached upon a vital part, and from the ravages of which he gradually sank. For five years the apparatus had done service.

There was a second apparatus made use of in that case, but I was unable to obtain possession of it, as it was buried with the patient, having been retained in its position so as to better preserve the appearance of the corpse, for the benefit of his friends who desired to take a last view of the deceased.

I wish to say further that, in vulcanizing hard rubbers such as Dr. Gibson has shown you to-night, the rubber becomes very dense and may be worn for a number of years without absorption or the fluids of the mouth having the slightest effect on it. There may be absorption of the soft tissue by continual friction of the edges on account of these not being modelled to the form of the muscles.

In regard to the metallic clasps, I would object to any metal being attached to the natural teeth, inasmuch as the abrasion would end in making the teeth very sensitive and spoiling the margins. I would rather make the separation and have a continuous rubber ring. Thereby you will have no friction as compared with what you would have by a hardened substance coming in contact with the tooth structure. The moulding made of this material is now, I think, simplified for us, inasmuch as Dr. Teague, of South Carolina, has given us an impression material out of which you can drive the moisture and by means of which you can cast your die immediately after the impression is taken from the mouth. I think with that and with the insight that Dr. Gibson has given us into the use of these various appliances, we can, in many of the cases brought before us, help our patients to a thorough, useful, and scientific adaptation of the dental obturator.

*Dr. R. B. Winder.*—I have been pleased and gratified by the essay with which we have been favored by Dr. Gibson. I do not think that any adverse criticism or comment can properly be made upon that essay. My confidence is such in the experience that Dr. Gibson has had in this direction that I would not, if I could, criticise the successful work which has crowned his efforts after so many years of application on his part. I have had isolated cases of the class referred to, but, as Dr. Gibson has pursued this specialty for many years, I am disposed to accept what he says as facts that are not easily controverted. I would like to ask the *modus operandi* by which the impression of a soft palate is obtained, this question having special reference to the pencilling of the models.

*Dr. Gibson.*—The method employed is to add to the model already obtained successive layers of plaster of Paris applied with a camel's-hair brush, guided by looking into the mouth from time to time, until a complete cast is obtained, much in the same way as the artist models a head or hand.

*Dr. W. A. Mills.*—I would ask Dr. Gibson what method he adopts in order to prevent the plaster from setting too quickly?

*Dr. Gibson.*—I make use of the plaster only in small quantities. I place a certain quantity of plaster in a saucer and, as I proceed, I add water to the plaster. If that which I am using becomes stiff I discard it and use fresh plaster. The setting of the plaster can be retarded by applying vinegar to it. Molasses or sugar, when used in the mixing of plaster of Paris, will eventually make the plaster much harder than it would be if they were not used.

*Dr. A. J. Volck.*—I have been entertained and instructed by the address of Dr. Gibson, and much pleased with the illustrations he has given. I may say that, in the early part of my practice, many years ago, I had a number of these cases on hand. In 1851 I had one such case in connection with Dr. Hollahan, of Wheeling, W. Va., whose name, I suppose, is familiar to all of the gentlemen present. An apparatus which we then made was regarded by us as a very great thing, but I am apprehensive that if it were exhibited to-day it would be looked upon as somewhat antiquated and perhaps as a relic of antediluvian times. Its method of construction was simply this: A pulp was formed close to the posterior naris. This was connected with the plate by spiral springs and slides. At the same time a nose was made, which were held upon the face by a big pair of spectacles, with a bar running through the nose and a spiral spring to hold the lower part of the nose back against the face. When the machine was all put together it looked quite well, but the patient felt very much as did one of the old knights in harness, perfectly safe but unable to move.

I was exceedingly glad to hear Dr. Gibson say what he did, because he has given me several new ideas. I am well satisfied, from my own experience, that Dr. Gibson is correct when he says that the taking of an impression of the soft palate is very difficult. I have tried it over and over again but always failed. In one of these cases, which was that of a small boy who is now only eighteen years of age, I attempted to cover a little over the hard palate, but as it irritated the palate I shaved it off until that irritation wore away. The boy is wearing that plate now, and his condition is very much improved. As he is young and still growing, it is almost impossible to make a plate that he can wear for any length of time. I expect to be obliged to make another plate for him in the course of a year.

There is one matter spoken of by Dr. Gibson about which I would like to say a word, especially as it is in point. I refer to his allusion to hare-lip. It recalls to my mind a case in which I operated upon a patient and in which the operation was very successful,—that is to say, scarcely any deformity in the face is apparent. But I have found, in that case, a couple of incisors growing inside. In operating on hare-lip, the maxilla being entirely divided, I took those parts and pressed them together. They healed perfectly and the lip is perfect, although you can see the scar that remains. But now here come these teeth. They have grown out and seem to be either detached from each other, or not in the bone at all; that is, they move about without being loose in the gum. Of course there must be some deformity about the roots. The question in my mind now is as to whether I had better extract these teeth now, because I do not know what the result is to be, or whether I should wait until the other teeth make their appearance in his jaw and perform the operation then. I would be glad to have the opinion of Dr. Gibson as to the proper course to pursue.

*Dr. Gibson.*—If those teeth are going to interfere with the articulation, I would extract them.

*Dr. Volck.*—They do not at present.

*Dr. Gibson.*—Then I would let them alone for the present.

*Dr. Volck.*—The fact is this: they are not growing flat with the face, but are growing sideways.

*Dr. Gibson.*—Will they ever be of any service?

*Dr. Volck.*—No.

*Dr. Gibson.*—Then I would extract them.

*Dr. Volck.*—That is about what I had determined to do.

*Dr. Palmer*, of New York.—My experience with this subject in a practical way is somewhat limited, as it is one that rarely comes under my observation. Much that has been said here was full of information for me, and I can say that a doctor, like men of other professions, must go from home to hear the news. I can only reiterate what Dr. Winder has said, that, knowing comparatively little of the subject from personal observation, I am content to accept as facts the statements that have been made by one who has given the matter so much care and attention as have been given to it by the essayist. I think that those of us who know anything of the use of vulcanite can thoroughly understand why Dr. Gibson has been so successful in the process of inflating those attachments.

*Dr. S. H. Guilford*, of Philadelphia.—I did not hear the paper which was read, and therefore cannot make any remarks directly upon it. I have had very little experience, as very few cases in that line have come to me. I am rather glad of this, for I think it certainly must be very difficult to do work successfully in many of such cases. It has occurred to me to mention a case that was sent to me quite recently by a surgeon in Philadelphia. It was the case of a lady who had lost her nasal bones. I think they had been removed entirely by an operation. The skin over the space had been drawn very tightly, and there was some adhesion in the centre where the skin had gone in. The face was but slightly disfigured. The falling in could be noticed, but the previous operation had been so nicely performed that the imperfection was not conspicuous. The surgeon communicated with me to ascertain whether I could not make an artificial piece from what he called "some unctious material," to supply the place of the nasal bones, and which he proposed to insert by an operation. I informed him that, in my judgment, this was entirely inadvisable because the pressure of the skin, which was then very tense, would be made greater after being drawn up to cover the artificial piece, and that that pressure would cause resorption of the bone upon which it rested and also of the soft tissues adjacent.

I confess that I was at the time somewhat surprised by the intimation conveyed by the question that surgeons apparently know so little about matters connected with dentistry. I would have supposed, in that case, that the surgeon was better informed in regard to the resorption of bone and understood that the tense skin would not bear any additional tension. But this is not germane to the essay nor to the subject.

*Dr. George Evans*, of New York.—I have had very little experience in the specialty treated of by Dr. Gibson, and the statement made by Dr. Guilford is about what I would have said if the call upon me had been made earlier. I have felt that I had not that experience which is essential to enable me to take hold of such cases and treat them effectively. The few that have come to me I have been generous enough generally to pass over to some member of the profession with more experience than myself, and whom I considered more competent than myself to take hold of them.

*Dr. A. J. Volck.*—One question that occurs to me I would like to address to the meeting generally, although it is one that has no immediate connection with cases like those which have been spoken of, but I mention it here, as this subject may not be again presented until our next meeting, and the inquiry is upon a matter about which it is of interest to us to know something.

We are occasionally called on to furnish artificial noses, and this occurs especially in cases where the deformity is the result of disease. My question is, What is the best material of which to make those artificial noses? I have seen them made of vulcanized rubber, I have seen them made of gutta-percha, I have seen them made of wax, I have seen them made of silver and of gold. If any gentleman present has had any experience in the matter, I would like him to give us the benefit of it.

*Dr. K. C. Gibson.*—I have had some little experience in making artificial noses and also in making artificial ears; and I am perhaps equally as desirous as the gentleman who asked the question to have information as to what substance should be preferred for that purpose. We have, so far, obtained better results in the use of celluloid than have been obtained in the use of any other substance of which I have any knowledge. Some years ago a celluloid nose was made for a gentleman, in a metal die, which was retained. A few months later that gentleman telegraphed for a duplicate nose, and some time later we received a letter from him stating that, while smoking a cigar, his nose caught fire and was burned. Perhaps he studied economy and retained the cigar in his mouth until it became so short that it came in contact with his nose. The liability to an accident of this kind is a serious objection to the use of celluloid. It might be advisable to use it in cases where the object is to create a prejudice against the smoking habit. The use of tobacco in syphilitic cases, as is well known to the medical profession, is very detrimental to the efforts of the physician, and, consequently, the use of celluloid might be advocated as a means of compelling the patient to discontinue the use of tobacco.

You can take different colors of the celluloid, and by pressing it together and remoulding it, you can finally get a color which will closely resemble flesh color. But if a patient goes out on a cold day, his face may redden while his nose retains the original color, and under other circumstances the reverse may be the case. It may be safe to assume that the use of liquor would not be likely to affect the appearance of a nose of that kind.

Seriously speaking, gentlemen, the question is one that should be solved. There is a class of unfortunate individuals who need our services and who are turned over to us by the medical profession. I wish we had some substance that would take the place of celluloid.

In connection with that matter, gentlemen, I wish to say that one of the models which I have exhibited, and which has been handed around to you, is the one in which the patient had used cotton for the plug. In this case the man had also lost his nose. He was much pleased with the result, in having his articulation restored; and, although sixty-odd years of age, he was very anxious to improve his appearance. Four or five months after the introduction of the obturator, he desired to have a nose made. This was made for him by taking a flap from the forehead. It was what is called a plastic operation. It resulted, however, in the death of the poor fellow from pyæmia.

Let me say here that in those cases which result from syphilis, great care is to be exercised by the dentist in adjusting the nasal substitute. Where the deformity is due to an accident, a gunshot wound or a similar cause, the tissue will tolerate a fixture of that kind; but where the deformity is due to disease, and you are obliged to sustain the appliance on springs resting on the lower part of the nasal cavity, if you are not very careful with it, there will be, after the lapse of a few months, absorption and an aggravation of the disease, and you will wish you had never made a nose for the poor patient.

*Dr. D. Genese.*—I wish to say that there is one substance which has not been mentioned by either gentleman, and that is, a platinum base with porcelain, which with a little care and a few extra firings may be built up to form a nose. This can be matched to nearly a flesh color, and the glaze may be taken off by the application of hydrofluoric acid or it may be ground off. It is better to take it off with the acid, as this gives a certain appearance of dulness, and it may be polished with Arkansas stone. In burning, you can make it of whatever shape you prefer, and may keep it in its shape by the use of flaps of soft platinum, and it may be formed in any way you want it by making the edges of it of round wire, solid, of fine gold. It is necessary that, in work of that kind, especial care should be taken to avoid accidents from dropping it, although accidents are of course liable to occur under any circumstances. About two years ago I put on one which was of rubber. I used chloroform and gum rosin with the water colors with which I colored it, after mixing these together. In this way I got a beautiful imitation of the flesh color. But the nose needed recoloring every month. I think Dr. Winder saw that nose. It is still being worn by the patient for whom it was made. I anticipate having my attention again called to it at an early day.

*Dr. R. Grady.*—A gentleman present is desirous of learning from Dr. Genese how the water colors stand the rain.

*Dr. Genese.*—Instead of mixing with water, as I have explained, I mixed the colors with chloroform and powdered gum. This formed an impervious coating.

*Dr. A. J. Volck.*—And it was one that would not wash off.

I wish to say that my motive in asking the question, which I propounded some moments ago, was to acquire information on the subject from the members generally. In reference to what has been said, I may say that I do not believe in the usefulness of platinum noses, because of their weight. They are extremely heavy. If we are to use metal at all, we may use gold and carefully finish up the work. If in the patient's family there is a nose which the artificial one can be made to resemble, or if there is a distinctive family nose in a particular case, we imitate that nose. I do not think that, in cases generally, clamps inside of the nose can be made use of. This is especially true in those cases in which the loss of the nose has been caused by disease, and I have found that this is quite generally the cause. My plan has been to model the nose on the face and then form it in gold, after which I paint it with Japan colors, which are afterwards baked into the model and which retain their color for a long time very well. I do not use water colors for this purpose. I make the color of the nose a little stronger than that of the face, as otherwise the nasal appendage is apt to look too pale. If a patient afterwards returns you a nose that has been painted according to some definite plan in this way, you are able to determine from memory how that nose should look. If the patient has a pair of these noses, so that he is able to change them about, he can always have a clean nose and one that is in good order by having it repainted and repaired.

I have also prepared a nose of common, clean vulcanized rubber for use by a lady patient. It was painted up with common oil paints. The lady paints it herself, and is quite an artist. She has become so expert in painting her own nose that it is no more difficult for her to do it than it is for her to wash her face. She touches it up in the morning. It dries very quickly, and she always has a good nose.

